# 

**Published:** 1858-11

**Authors:** 


### RECEIPTS FOR JOURNAL UP TO DECEMBER 10th, 1858.

### Illinois.

| | | |
|---|---|---|
| Dr. John Emery, | vol. 1, 1858, | $2 |
| " J. W. Floyd, | " " | 2 |
| " L. P. Cheney, | " " | 2 |
| " G. A. Byrnes, | " " | 2 |
| " H C. Daniels, | " " | 2 |
| " E. Vogeler, | " " | 2 |
| " R. F. Price, | " " | 1 |
| " James Leeds, | " " | 2 |
| " J. H. Wilkinson, | " " | 2 |
| " J. G. Tilden, | " " | 2 |
| " Hosea Davis, | " " | 2 |
| " H. T. Bartlett, | " " | 2 |
| " J. P. Terrell. | " " | 2 |
| " J. B. Samuel, | " " | 2 |
| " Samuel McNair, | " " | 2 |
| " J. Van Dyke, | " " | 2 |
| " John Wright, | " 6, 1857, | 2 |
| " R. H. Gibson. | vols. 6, 1, 2, 1857–8–9, | 6 |
| " H. J. Van Winkle, | vols. 1, 2, 1858–9, | 4 |
| " S O. Long, | vol. 2, 1859, | 2 |
| " W. R. Griswold, | " " | 2 |
| " J. R. Webster, | " " | 2 |
| " J. S. Mayfield, | " " | 2 |
| " N. O. Pearson, | " " | 2 |
| " L. D. Martin, | vols. 6 & 1, 1857–8, | 3 |

### Wisconsin.

| | | |
|---|---|---|
| Dr. J. H. Leitch, | vol. 1, 1858, | $2 |
| " R. W. Earll, | " " | 2 |
| " Jas. Cady, | vols. 6 & 1, 1857–8, | 4 |
| " H. Warne, | vol. 2, 1859, | 2 |
| " E. G. Dyer, | " " | 2 |
| " J. H. Cooper, | " " | 2 |

### Indiana.

| | | |
|---|---|---|
| Dr. Alphonso Wood, | vol. 1, 1858, | $2 |
| " W. H. Ball, | " " | 2 |
| " A. O. Miller, | " " | 2 |
| " Samuel Ross, | " " | 2 |
| " J. H. Spurrier, | " " | 2 |
| " J. W. Green, | " 2, 1859, | 2 |
| " Carroll & Murray, | " " | 2 |
| " J. R. Congdon, | vols. 4. 5 & half of 6, 1855–6— | 5 |

### Michigan.

| | | |
|---|---|---|
| Dr. L. D. Tompkins, | vol. 1, 1858, | $2 |
| " S. Stoddard, | vols. 4, 5, 6, 1 & 2, 1855, 6, 7, 8 & 9, | 10 |

### Arkansas.

| | | |
|---|---|---|
| Dr. S. L. Urmston, | vol. 1, 1858, | $2 |

### California.

| | | |
|---|---|---|
| Dr. M. R. Fowler, | vol. 2, 1859, | $2 50 |

### Minnesota.

| | | |
|---|---|---|
| Dr. H. L. Coon, | vols. 5, 6 & 1, 1856, 7 & 8, | $6 |

### Iowa.

| | | |
|---|---|---|
| Dr. J. Muncey, | vol. 1, 1858, | $2 |
| " A. B. Ireland, | " " | 2 |
| " H. Fisk, | " " | 2 |
| " J. H. Griffith, | " " | 2 |
| " R. L. Hallock, | vols. 6 & 1, 1857–8, | 4 |
| " J. H. Graham, | " " | 4 |

